# Partial endocardial cushion defect combined with persistent left superior vena cava to left atrial roof & cor triatriatum in an adult patient

**DOI:** 10.1186/1749-8090-8-S1-P93

**Published:** 2013-09-11

**Authors:** WK Baek, YS Kim, YH Yoon, JT Kim, HK Shinn, DH Kim

**Affiliations:** 1 Department of Thoracic and Cardiovascular Surgery, Inha University Hospital, Incheon, South Korea; 2 Department of Anesthesiology, Inha University Hospital, Incheon, South Korea; 3 Division of Cardiology, Department of Internal Medicine, Inha University Hospital, Incheon, South Korea

## Background

Completely unroofed coronary sinus associated with persistent left superior vena cava (PLSVC) is accompanied in about 3% of patients with partial endocardial cushion defect(p-ECD).

## Methods and results

We report here with a case corresponding to the above disease entity, in which pulmonary veins were drained into a separate posterior chamber, which was connected to the common atrium, thus forming cor triatriatum. A 42-year-old Asian female was consulted for operation of p-ECD and PLSVC, which were detected during the evaluation of complete heart block. She complained of worsening dyspnea and syncope attack for recent one year. The PLSVC had no connection with right sided SVC and was thought to be drained into the left atrial roof directly. The operation was carried out under standard hypothermic cardiopulmonary bypass. A large common atrium without septal remnant was encountered on opening right atrium. The coronary sinus opening was absent and PLSVC was directly drained into atrial roof, to the right of the left atrial appendage. An opening measuring 3x3cm, which at first was confused as atrial septal defect, was seen to the right of PLSVC. An accessory chamber, draining all four pulmonary veins was connected to the common atrium by this opening. After closing mitral valve cleft, a generous intraatrial patch made of bovine pericardium was used to partition the common atrium. The opening connecting pulmonary venous chamber to the atrium was widely extended inferiorly and the upper part of the opening was partly closed to minimize distortion of the patch. The left atrial appendage was also incorporated into the right side of patch to simplify the baffling procedure. The tricuspid annuoplasty was carried out and permanent epicardial pacemaker was implanted n the end. The patient recovered without events.

## Conclusion

The mitral and tricuspid regurgitation were corrected on follow-up echocardiography. The intraatrial baffle seemed to be intricate, but there were no residual shunt or flow obstruction.

